# Editorial Comment: Continuous monitoring of intrapelvic pressure during flexible ureteroscopy using a sensor wire: a pilot study

**DOI:** 10.1590/S1677-5538.IBJU.2021.01.06

**Published:** 2020-11-18

**Authors:** Alexandre Danilovic

**Affiliations:** 1 USP Faculdade de Medicina Hospital das Clínicas São PauloSP Brasil Serviço de Urologia, Hospital das Clínicas da Faculdade de Medicina da USP - HCFMUSP, São Paulo, SP, Brasil.

Doizi S ^1^ ^2^, Letendre J ^3^ ^4^ ^5^, Cloutier J ^3^ ^4^ ^6^, Ploumidis ^3^ ^4^ ^7^, Traxer O ^3^ ^4^

## COMMENT

Flexible ureteroscopy (fURS) relies on endoscopic vision that depends on fluid irrigation. The intrapelvic pressure (IPP) reached during flexible ureteroscopy (fURS) is a matter of great concern because high levels may cause pyelovenous backflow and fornice rupture (1). In vitro study demonstrated all irrigation systems generate high levels of pressure (2). Recently, other authors compared two automated irrigation systems using an in vitro ureteroscopy model and although both systems provided steady irrigation at safe pressures, the measured IPP exceeded the desired settings across the entire tested range (3). This imprecision is potentially dangerous. Unfortunately, the surgeon cannot sense IPP and we lack practical means to measure IPP during fURS.

Doizi et al. evaluated, in a pilot study, the feasibility of measuring the IPP during fURS using a wire with a pressure sensor. The device used to measure IPP was a 0.014” wire routinely used by cardiologists to assess fractional flow reserve in coronary arteries. The device transmits the pressure signal and temperature instantly. Constant irrigation pressure set at 80 cmH2O and on-demand forced irrigation by a hand held pump was used during fURS. The authors were able to observe very high levels of IPP during fURS and two patterns of IPP during on-demand forced irrigation. Rapid forced irrigation caused peaks on IPP but never returned to baseline. Long forced irrigation generated long plateau on IPP correspondent to the force applied. Of note, the authors used the pressure wire as a safety guide wire and were able to use it to place over a silicone double J.

The impact of high IPP on clinical outcomes is not completely known. Despite advice to do the opposite, many surgeons use devices that generate high levels of pressure. However, reported complications of fURS as increase in creatinine, bleeding, infection and subcapsular hematoma are very low (1, 4). Ureterorenoscopy procedure may cause harmful early term effect to the kidney evidenced by increase of inflammatory markers in urine but the effect seems to disappear over time (5). It may depend not only on the level of IPP but also on how much time high pressures are applied to the collecting system. Also, patient and collecting system features may play important role (6). The impact of IPP should be evaluated not only during fURS but also during percutaneous nephrolithotomy, where it has been associated with infection in experimental study (7). Therefore, an efficient way to monitor IPP is welcome to help evaluate clinical outcomes.
